# Desmoid tumor suggesting recurrence of retroperitoneal liposarcoma: case report and review of the literature

**DOI:** 10.1097/RC9.0000000000000530

**Published:** 2026-05-15

**Authors:** Rayan Aribi, Matthias Tallegas, Mehdi Ouaissi, Urs Pabst-Giger, N. Michot

**Affiliations:** a Department of Digestive, Oncological, Endocrine and Hepatobiliary Surgery and Liver Transplantation, Trousseau Hospital, C.H.U de Tours, Tours, France; b Department of Anatomopathology, Trousseau Hospital, C.H.U de Tours, Tours, France; c UMR1327 ISCHEMIA Membrane Signalling and Inflammation in Reperfusion Injuries, Université de Tours, Tours, France; d Fliedner University of Applied Sciences, University of Applied Sciences Düsseldorf, Düsseldorf, Germany

**Keywords:** biopsy, desmoid tumor, recurrence, retroperitoneal liposarcoma

## Abstract

**Introduction:**

Liposarcomas are rare malignant tumors of adipocytic origin with a high propensity for local recurrence. Although desmoid tumors are benign, they may present with clinical and radiological features similar to those of soft tissue sarcomas, which may complicate the differential diagnosis.

**Presentation of case:**

We present the case of a patient who underwent an initial resection of a retroperitoneal liposarcoma. During routine follow-up, imaging suggested a local recurrence. Following a multidisciplinary discussion, a second surgical intervention was performed, revealing histopathological features consistent with a desmoid tumor rather than a recurrent liposarcoma.

**Discussion:**

This case demonstrates that a lesion initially suspected to be recurrent liposarcoma may actually be a desmoid tumor. It highlights the importance of a thorough multidisciplinary evaluation and histological confirmation prior to planning further treatment, given that management strategies differ significantly between these conditions.

**Conclusion:**

This observation highlights that lesions following abdominal liposarcoma resection do not always represent tumor recurrence, and that biopsy remains essential for accurate diagnosis and optimal management.

## Introduction

Liposarcomas are rare malignant mesenchymal tumors representing 15–20% of adult soft tissue sarcomas. They tend to occur in deep anatomical locations^[^1^]^. The standard treatment is complete surgical excision with negative margins (R0), which is performed in specialized centers within the French Sarcoma Network (NetSarc+) in accordance with national and European guidelines^[^2,3^]^.

HIGHLIGHTS

- This is the first description of a desmoid tumor mimicking a recurrence of retroperitoneal liposarcoma, along with a discussion of its diagnosis, care, and treatment.
- It emphasizes the importance of multidisciplinary discussion and expert care in an expert center.

Despite optimal surgery, local recurrence remains frequent, particularly for well-differentiated tumors, those in deep locations, large tumors, and poorly differentiated histology^[^4–6^]^. Postoperative follow-up typically involves long-term clinical and radiological monitoring, often with MRI or CT scans, and can extend up to 10 years. This is to detect recurrences and late complications such as fibrosis, chronic pain, and neurological and digestive sequelae^[^7^]^.

Recurrences require multidisciplinary evaluation, with surgery and/or radiotherapy as indicated. The STRASS trial^[^8^]^ did not demonstrate the overall benefits of preoperative radiotherapy for retroperitoneal sarcomas, but it did suggest potential advantages for certain cases of liposarcoma, particularly those involving preoperative recurrence.

Desmoid tumors, although histologically benign, can resemble sarcomas. They arise in deep tissues, exhibit infiltrative growth, and recur locally, sometimes following surgery^[^9,10^]^. Several reports describe cases where desmoid tumors were initially suspected to be sarcoma recurrence but were later confirmed by biopsy^[^11,12^]^.

Recurrence of retroperitoneal sarcoma is well-documented. However, distinguishing true recurrence from other postoperative lesions remains a challenge. To our knowledge, abdominal desmoid tumors occurring after retroperitoneal liposarcoma surgery have not previously been reported. The aim of this report is to describe a patient in whom a mass suggestive of recurrence was detected during follow-up, which was subsequently confirmed to be desmoid fibromatosis. This case highlights the importance of biopsies in cases of suspected relapse and illustrates the challenges involved in the postoperative surveillance of patients with liposarcoma.

This case report has been presented in line with the SCARE checklist^[^13^]^.

## Presentation of case

**Figure 1.**
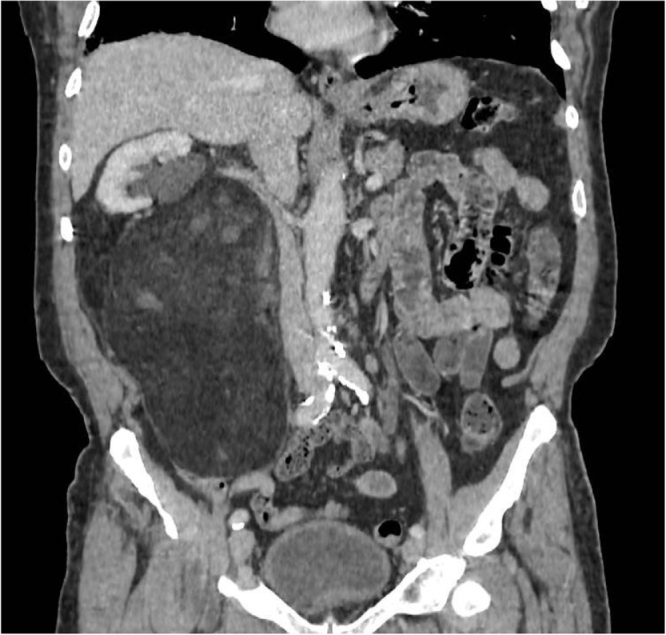
Preoperative CT scan of right liposarcoma.

In April 2023, a large retroperitoneal lesion was incidentally found during a chest CT scan performed on an 81-year-old patient with no significant medical history who was experiencing intermittent rib pain (Fig. 1). An abdominal MRI scan confirmed the presence of a heterogeneous, fatty, 19 cm retroperitoneal mass on the right, with no involvement of the superior mesenteric vessels (Supplemental Digital Content Figure 1, available at: http://links.lww.com/IJSCR/A52).

Following a multidisciplinary review in July 2023, a percutaneous CT-guided biopsy was recommended. The biopsy, performed in October 2023, confirmed a well-differentiated liposarcoma with MDM2 amplification. A follow-up CT scan in November 2023 showed that the tumor size had remained stable. Surgical excision was subsequently planned.

**Figure 2.**
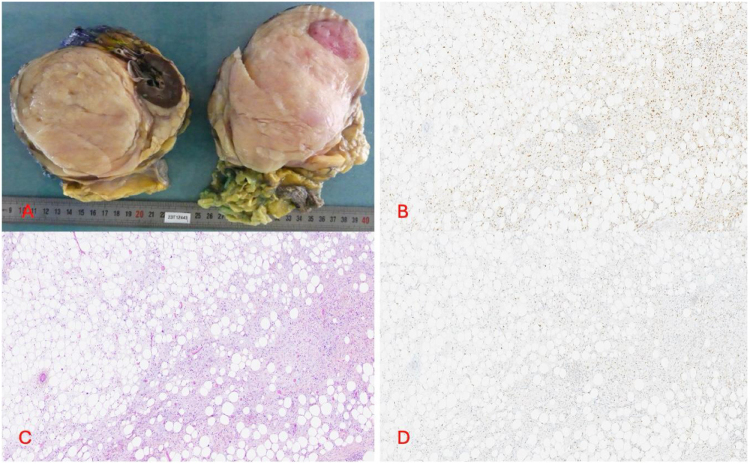
Liposarcoma resection specimen. (A) Macroscopic view; (B) HPS: Hematoxylin-eosin-saffron staining, ×100 magnification, highlighting the lipogenic differentiation of the retroperitoneal tumor, composed of a mixture of mature adipocytes, lipoblasts, spindle cells and pleomorphic cells on a fibromyxoid background (lipomatous and sclerosing subtypes); (C) HMGA2 immunostaining, clone IF2, VITRO MASTER DIAGNOSTICA, ×100 magnification; and (D) MDM2 immunostaining, polyclonal, BIOCHECK, ×100 magnification: Tumor cells overexpressed HMGA2 and MDM2 due to the canonic 12q14-q15 genomic amplification.

In December 2023, the patient underwent a median laparotomy with a right transverse split and a compartmental resection, including the right colon, right kidney, and right adrenal gland. Histopathological examination revealed a 30 cm well-differentiated retroperitoneal liposarcoma (lipoma-like and sclerosing subtypes) with complete resection (R0m) and submillimeter posterior margins. The tumor abutted the right kidney and ureter but showed no infiltration, and the resected organs were uninvolved (Fig. 2). Tumor cells exhibited lipogenic differentiation, comprising mature adipocytes, lipoblasts, spindle cells, and pleomorphic cells on a fibromyxoid stroma. There was HMGA2 and MDM2 overexpression due to canonical 12q14–q15 amplification.

Postoperative follow-up was initiated according to multidisciplinary recommendations. In November 2024, a CT scan revealed a 4.6 cm mass in the right mesentery, suggestive of recurrence, which had grown by January 2025. As vascular and digestive involvement precluded biopsy, surgical resection was performed in March 2025 via midline laparotomy, involving segmental colonic resection and ileocolic anastomosis (Supplemental Digital Content Figure 2, available at: http://links.lww.com/IJSCR/A52). Surgery was performed following multidisciplinary discussion in our specialized center within the NetSarc+ network.

Histopathological examination revealed a 9 cm spindle-shaped tumor consistent with deep fibromatosis (desmoid tumor). This tumor was characterized by low cellularity, fasciculated architecture, a scar-like stroma, and an absence of lipogenic or pleomorphic elements. Tumor cells were negative for HMGA2 and MDM2 (see Supplemental Digital Content Figure 3, available at: http://links.lww.com/IJSCR/A52).

Postoperative recovery was uneventful, and the patient was discharged on the eighth day after surgery. At the 8-month follow-up, there was no evidence of recurrence.

## Discussion

This case illustrates the diagnostic and management challenges posed by the suspected recurrence of retroperitoneal liposarcoma, which may mask a distinct histopathological entity. Surveillance imaging detected a new intraperitoneal mass that was difficult to biopsy due to its proximity to vascular and digestive structures.

Recurrence of retroperitoneal liposarcomas is common, often necessitating repeat surgical intervention. Desmoid tumors, however, can arise in post-surgical sites, likely triggered by mechanical or inflammatory stimulation of tissues. Similar cases have been reported in limb and neck regions, highlighting the predilection of desmoid tumors for scar tissue and prior surgical sites^[^10^]^. Imaging alone is often insufficient to distinguish desmoid fibromatosis from sarcoma recurrence, particularly for poorly vascularized, tissue-based masses in close contact with critical structures.

Current guidelines favor active surveillance for desmoid tumors, tailored to clinical presentation and disease progression, as recommended by ESMO (European Society for Medical Oncology)^[^14^]^. Surgical resection is reserved for symptomatic or progressive lesions. Systemic therapies – including tyrosine kinase inhibitors (e.g., sorafenib), hormonal therapy, or chemotherapy – may be considered for inoperable or recurrent cases.

In this case, surgery was chosen because the mass was inaccessible to biopsy, the patient’s history suggested recurrence, and the procedure was considered low-morbidity. Following multidisciplinary discussion, the options were limited to either radiological monitoring without histological confirmation or definitive surgical excision. As recommended^[^2^]^, the surgery was performed following the compartmental strategy to avoid tumoral rupture during the procedure.

**Figure 3.**
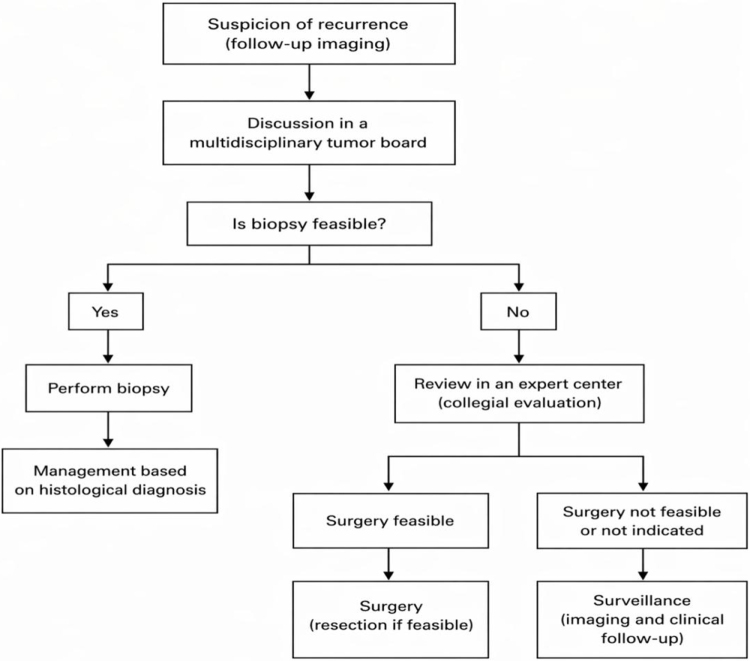
Proposed management algorithm for a suspected recurrence of retroperitoneal liposarcoma.

This report underscores the importance of integrating clinical, radiological, and histopathological data within a multidisciplinary framework to guide optimal management (Fig. 3). The inability to obtain a preoperative biopsy represents the key limitation. It highlights the importance of a multidisciplinary evaluation with an expert sarcoma network such as NetSarc+/Réseau de Référence en Pathologie des Sarcomes des tissus mous et des viscères, like our specialized center.

It further emphasizes the critical role of biopsy, whenever feasible, to avoid misdiagnosis and unnecessary or inappropriate interventions.

## Conclusion

This case highlights the importance of including desmoid tumor in the differential diagnosis of a mass suspected to be a recurrence of liposarcoma. Wherever possible, a biopsy is essential to confirm the diagnosis histopathologically and guide appropriate management.

To our knowledge, this is the first reported case of an abdominal desmoid tumor arising after surgery for retroperitoneal liposarcoma. The report emphasizes the crucial role of expert multidisciplinary evaluation in determining the best course of action for patients and avoiding unnecessary procedures when pathology can be obtained preoperatively.

## Data availalability statement

The datasets generated and/or analyzed during the current study are available from the corresponding author on reasonable request.
